# Major infectious diseases affecting the Afghan immigrant population of Iran: a systematic review and meta-analysis

**DOI:** 10.4178/epih/e2015002

**Published:** 2015-01-07

**Authors:** Behzad Pourhossein, Amin Doosti Irani, Ehsan Mostafavi

**Affiliations:** 1 Department of Epidemiology, Pasteur Institute of Iran, Tehran, Iran; 2 Department of Virology, School of Public Health, Tehran University of Medical Sciences, Tehran, Iran; 3 Department of Epidemiology and Biostatistics, School of Public Health, Tehran University of Medical Sciences, Tehran, Iran; 4 Research Centre for Emerging and Reemerging Infectious Diseases, Pasteur Institute of Iran, Akanlu, Kabudar Ahang, Hamadan, Iran

**Keywords:** Afghan, Immigrant, Infection, Health, Systematic review, Iran

## Abstract

**OBJECTIVES:**

As Afghans make up the largest group of foreign nationals in Iran, the aim of this study was to assess the proportion of Afghan immigrants among those afflicted by the most prevalent infectious diseases in Iran.

**METHODS:**

National and international online scientific databases were searched through November 2013. The reference lists of included studies were also searched. All descriptive studies concerning the most common infectious diseases in Iran, including tuberculosis, multiple-drug-resistant tuberculosis, malaria, cholera, Crimean-Congo hemorrhagic fever, leishmaniasis, and hepatitis B were retrieved. The nationality of patients was not considered. The selection of studies and data extraction was performed separately by two authors. Results were reported using a random effect model with a 95% confidence interval (CI).

**RESULTS:**

The overall proportion of Afghan immigrants with the aforementioned infectious diseases was 29% (95% CI, 21 to 37). According to a stratified analysis, the proportion of Afghan immigrants afflicted with tuberculosis was (29%), multiple-drug-resistant tuberculosis (56%), malaria (40%), cholera (8%), Crimean-Congo hemorrhagic fever (25%), leishmaniasis (7%), and hepatitis B (14%).

**CONCLUSIONS:**

It is highly recommended to monitor the health status of the Afghan immigrants when entering Iran, to reduce the spread of communicable diseases, which are viewed as serious in international health regulations.

## INTRODUCTION

According to statistics released by the United Nations in 2013, an estimated 232 million people were classified as immigrants. This amounts to approximately three percent of the world’s population [1]. Illegal immigration is considered a punishable crime by many governments [2]. Illegal immigrants, also referred to as undocumented immigrants, constitute, under certain conditions, a refugee population in a host country. Since reliable statistics are not available regarding undocumented immigration, it can only be estimated that about twenty to thirty million illegal immigrants exist worldwide [3]. Afghans account for the majority of the immigrants in Iran [4].

After Iraq and Turkmenistan, Afghanistan shares the longest border with Iran. Due to the occupation of Afghanistan by the Union of Soviet Socialist Republics between 1980 and 1989 and subsequent internal wars in Afghanistan, around 2.9 million Afghans have immigrated to Iran [5]. Over recent years, although a myriad of encouragements as well as threats have been presented to these nationals to return to Afghanistan, the repatriation process has been extremely slow. Poverty, unemployment, and a lack of security in Afghanistan are among the most important factors that prevent Afghans from returning to their own country. The Iranian government began implementing plans for Afghans’ gradual return in 1995 after the exit of the Soviet Union from Afghanistan. In a census conducted in 2001, 2.5 million foreign nationals were counted, and by 2007, this figure had decreased to one million. Of these, more than 940,000 were Afghan. However, this figure reflects the official statistics; the number of undocumented nationals is much higher [5,6]. Four hundred thousand Afghan residents are born in Iran and the mean duration of their residence is over fifteen years [7].

In spite of the longstanding presence of Afghan immigrants, they remain in the lower levels of social, political, and economic life in Iran. Afghan immigrants in Iran work predominantly in jobs requiring few or no skills [8].

The presence of these foreign nationals has imposed multiple challenges, such as new health risks, social problems, and cultural incompatibilities. Among these challenges, the spread of infectious disease is a significant problem [9,10].

Immigration can negatively affect the state of health of immigrants, because of the lack of availability of health services [11]. The Iranian government has practiced health surveillance and treatment of all foreign nationals regardless of their nationality. Immigration, particularly of Afghans, has been associated with an increase in the number of reports of some diseases in Iran. Since the first wave of Afghan immigrants came to Iran (more than 30 years ago), immigrants have had access to essential health care and, education services [12].

According to the World Health Organization (WHO) report, the burden of most infectious diseases, including tuberculosis (TB), leishmaniasis, and malaria, in Afghanistan is greater than in Iran [13-15].

Several studies concerning infectious diseases conducted in Iran have addressed the proportion of Afghan immigrants among the patients. However, results have been inconsistent, and thus there is no accurate estimation of Afghan immigrants with infectious diseases in Iran. The aim of this meta-analysis was to estimate the overall proportion of Afghan immigrants with infectious diseases in Iran.

## MATERIALS AND METHODS

To conduct this study, the list of all major infectious diseases that Afghans could potentially be infected with was drawn up. This list was extracted via a web-based search and a primary search of related articles. Using the Delphi method and after consulting with ten experts in the Ministry of Health of Iran, the infectious diseases of greatest importance were selected.

### Searching

The major national and international databases were searched using the following key words: emigrants and immigrants, refugees, Afghan, Iran, prevalence, incidence, tuberculosis, human immunodeficiency virus (HIV), acquired immunodeficiency syndrome, malaria, cholera, Crimean-Congo hemorrhagic fever (CCHF), hepatitis B, hepatitis C, and leishmaniasis. The international databases included the Web of Knowledge (January 1945 to November 2013), ScienceDirect (January 1823 to November 2013), Medline (January 1950 to November 2013), and Scopus (January 1973 to November 2013). Moreover, the following national databases were searched: Science Information Database (up to November 2013), MagIran (up to November 2013), IranMedex (up to November 2013), and Medlib (up to November 2013).

The reference lists of all included studies were searched in order to obtain additional articles. We also contacted the authors of the included studies.

### Criteria for including studies

All descriptive studies regarding the most significant infectious diseases in Iran, namely, tuberculosis, cholera, hemorrhagic fever, malaria, HIV/acquired immune deficiency syndrome (AIDS), hepatitis B, hepatitis C, and leishmaniasis, were retrieved irrespective of the nationality of the subjects and language of the study. The main point of interest was the proportion of Afghan immigrants with the listed diseases in Iran.

### Data collection and validity assessment

Two authors (Pourhossein, Doosti Irani) independently screened the title and abstract of all obtained studies and then reviewed the full texts of the retrieved studies that met the inclusion criteria of this review.

The authors were not blinded to the names of the studies’ authors and journals. All disagreements between the authors about the final selection of studies were resolved by negotiation with a third author (Mostafavi). The agreement rate of the two authors was 88.44%, and the kappa statistic for checking reliability was 76.47%.

The variables that were extracted for data analysis included: study design, year and location of the study, type of infectious disease, nationality of participants, sample size, number of Afghan patients, and number of Iranian patients.

Six items from the Strengthening the Reporting of Observational Studies in Epidemiology (STROBE) statement checklist [16] were selected and used for assessing the quality of reporting. They included the following: (a) describe the setting, location and related times; (b) describe the demographic characteristics of the people that participated in the study; (c) give the inclusion and exclusion criteria; (d) describe the methods for the measurement of the outcome; (e) present the key element of the study design; and (f) state how the study sample size was derived.

The studies that fulfilled all items were classified as high quality, studies that did not meet one item were classified as intermediate quality, and studies that did not fulfill more than one item were classified as low quality.

The studies with a small number of patients were excluded from this review. For this purpose, a minimum sample of 18 was considered the cut-off point for estimating the proportion of Afghan patients in Iran (assuming *p* to be 85% with a statistical power of 80% and significance level of 5%). Thus, the studies with a sample size <18 were not eligible for this study and excluded from analysis.

### Statistical methods

The measure of interest was the proportion of Afghan immigrants afflicted with infectious diseases including TB, multiple-drug-resistant tuberculosis (MDR TB), cholera, CCHF, malaria, hepatitis B, and leishmaniasis with a 95% confidence interval (CI). Statistical heterogeneity was explored using Cochran’s Q test and Higgins’ I2 statistic. The analysis was performed using the STATA version 11 (StataCorp, College Station, TX, USA). The random effects model was used with a 95% CI.

## RESULTS

### Description of studies

**Figure 1.**
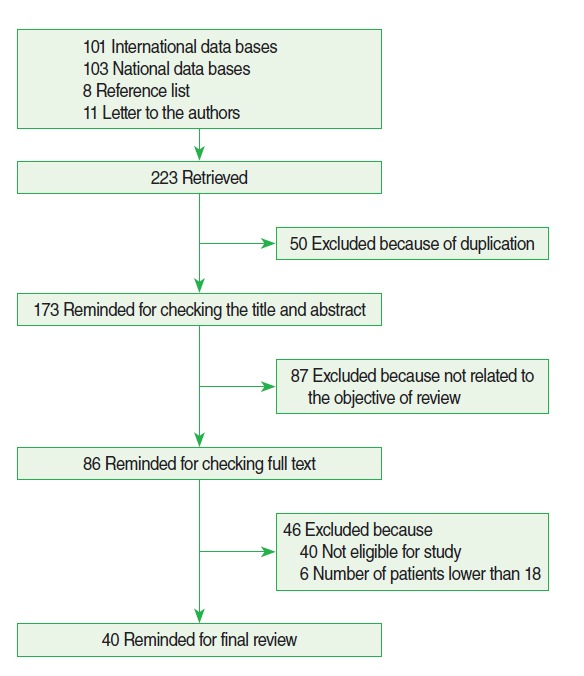
Flow diagram showing the phases of retrieving articles, checking eligibility criteria, and including the articles into the meta-analysis.

We extracted 223 studies up to November 2013, including 101 through searching the international databases, 103 through searching national databases, 8 through checking the reference lists of the included studies, and 11 through contact with the authors of the selected studies (Figure 1).

Of the 223 extracted studies, 50 studies were excluded because of duplication, 87 studies were excluded because they did not support the objective of this review, 40 studies did not meet the eligibility criteria for this review, and 6 studies had too small of a sample size. In the end we included 40 studies [17-56] in this review (Table 1). The combined included studies involved 125,248 participants.

We assessed the quality of included studies based on a STROBE checklist. Twenty-nine percent of included studies were high statement quality, 42% were intermediate quality, and 29% were low quality.

In this study, we performed a stratified analysis according to the quality of the studies, date of performance of the study, and type of infectious disease.

### Proportion of Afghan immigrants with tuberculosis and MDR tuberculosis in Iran

Twenty-two studies [19-21,23-26,31-34,36,37,41,44,46-50, 52,54] addressed the number of Afghan immigrants in Iran with TB from 1997 to 2010. The pooled proportion estimation of Afghan immigrants with TB on the basis of random effect was 29% (95% CI, 0.23 to 0.34). The lowest proportion, 4%, was reported by Ebrahimzadeh et al. [24] in 2009 and the highest proportion, 55%, was reported in the Kashan study [41] in 2009. Two studies addressed the number of Afghan immigrants with MDR TB [29,53]. The pooled proportion estimation of Afghan immigrants in Iran with MDR TB on the basis of random effect was 56% (95% CI, 0.34 to 0.77) (Table 2).

### Proportion of Afghan immigrants with malaria in Iran

Six studies [28,42,43,51,55,56] addressed the number of Afghan immigrants in Iran with malaria from 2003 to 2011. The pooled proportion estimation of Afghan immigrants in Iran with malaria on the basis of random effect was 40% (95% CI, 0.23 to 0.57). The lowest proportion, 4%, was in the Sistan and Baluchistan study [55] in 2011, and the highest proportion, 99%, was in the Rafsanjan study [56] in 2010 (Table 2).

### Proportion of Afghan immigrants with Crimean-Congo hemorrhagic fever in Iran

Two studies addressed the number of Afghan immigrants in Iran with CCHF [18,29]. The pooled proportion estimation of Afghan immigrants among patients with CCHF was 25% (95% CI, 0.20 to 0.30) (Table 2).

### Proportion of Afghan immigrants with cholera in Iran

Four studies addressed the number of Afghan immigrants among people in Iran with cholera [22,30,35,45]. The pooled proportion estimation of Afghan immigrants with cholera in Iran on the basis of random effect was 8% (95% CI, 0.004 to 0.16). The lowest proportion, 2%, was in a national study [30] in 2007, and the highest proportion, 19%, was in the Zabol study [35] in 2005 (Table 2).

### Proportion of Afghan immigrants with leishmaniasis in Iran

We found three studies concerning leishmaniasis that addressed the number of Afghan immigrants with leishmaniasis [17, 38,39]. The pooled proportion estimation of Afghan immigrant with leishmaniasis was 7% (95% CI, 0.00 to 0.13). The lowest estimate, 2%, was related by the Gorgan study [17] in 2004, and highest estimate was related by the Isfahan study [39] in 1994 (Table 2).

### Proportion of Afghan immigrants with hepatitis B in Iran

One study concerning hepatitis B had eligibility criteria for inclusion in this review [27]. The proportion of Afghan immigrants in Iran with hepatitis B reported in that study was 14%.

## DISCUSSION

TB, MDR TB, malaria, cholera, CCHF, leishmaniasis, and hepatitis B are the major infectious diseases among Afghan immigrants in Iran. Iran spends much annually for the prevention, control, and treatment of these infectious diseases. It has been estimated that the government spends more than 100,000 US dollars annually just to treat Afghan immigrants with TB [57, 58]. The results of this meta-analysis indicated that a high proportion of cases of serious infectious disease in Iran are Afghan immigrants; in fact, Afghan nationals comprised 29% of patients with infectious diseases in Iran. Furthermore our findings indicated that the infectious diseases with the highest proportion of Afghan immigrants were MDR TB (56%), malaria (40%), TB (29%), and CCHF (25%), in that order.

It should also be noted that the proportion of Afghan immigrants with infectious diseases in Iran has been increasing since 1994. This means that the high proportion of Afghan immigrants with infectious diseases is a critical public health issue in Iran. Health behavior and health care access factors may be related to the high proportion of Afghan immigrants in major infectious diseases in Iran. It has been noted that Afghan immigrants have little interaction with health care centers in Iran [59] and therefore do not receive adequate health care and treatment. This, in turn, facilitates the spread of infectious diseases in the community.

Afghanistan is known as a country with a high prevalence of TB. The latest reports indicate the annual incidence of TB in Afghanistan is 325 cases out of 100,000 members of the general population [60]. Over recent years, TB has been well controlled in Iran, but due to extensive migration of Afghans infected with TB, Iran is once again facing a spread of the disease [34]. The prevalence of MDR TB among the TB patients in Iran is reported to be 5.1% [25]. This meta-analysis found that 29% of TB patients and 55% of MDR TB patients in Iran are Afghan immigrants. Poor health conditions and services over the past three decades in Afghanistan have caused an increase in MDR TB among Afghans. Some of the immigrants to Iran lack a stable residence. This factor may cause a situation in which Iranian health service personnel cannot follow up during the treatment period [58,61].

One study conducted in Golestan province in north-east Iran also revealed that the incidence rate of all types of TB in Afghan families was significantly higher than in Persian families (42 and 17 cases in each 100 members of the population, respectively) [62]. A study in the city of Tehran carried out on 1,028 patients with TB showed that Afghan patients with pulmonary TB were younger than Iranian patients, and Afghan patients with a positive smear had a more severe type of the disease according to the degree of their sputum smear [58]. The proportion of Afghan immigrants in malaria cases in recent years in Iran was 40%. The reason for this high proportion may be due to the high incidence of malaria in Afghanistan—around 300 to 400 thousand cases yearly [63]. Frequent travel among foreign nationals and immigrants from eastern neighboring countries has become a major barrier to successfully controlling malaria in Iran [28]. A study in southeast Iran indicated that Afghan immigrants have significantly less knowledge of malaria transmission, and they have not implemented prevention practices to the extent of the general Iranian population. In addition, 37.9% of Iranians reported using bed nets, whereas only 10.3% of the Afghans did [59]. Previous experience has revealed that controlling the eastern borders and limiting extensive travel of Afghan nationals have been extremely effective in controlling the distribution of infectious diseases. Likewise, reported cases of malaria in Rafsanjan, in eastern Iran, were 62,000 in 2001, but this figure dropped to 29,000 in 2006 after the return of Afghans to their country [56].

CCHF is known to be a major endemic disease in Afghanistan. The results of this meta-analysis show that 25% of CCHF cases in Iran were in Afghan immigrants. The most important factor for this high proportion is that most cases of CCHF in Iran have been reported from Sistan and Baluchistan province, neighboring Afghanistan and Pakistan; most of the nationwide studies have noted trafficking of infected livestock from Afghanistan and Pakistan to be one of the main reasons for epidemics of this disease [64,65].

Our findings indicated 8% of cholera patients in Iran were Afghan immigrants. Afghanistan is one of the six countries accounting for 80% of all reported cases of cholera worldwide [66]. Therefore, it appears that Afghan immigrants may play a role in the initiation of cholera outbreaks in Iran [30].

The proportion of Afghan immigrants in Iran with leishmaniasis in this review was 7%. Infected humans can act as a reservoir for this disease. According to a report published by the WHO in 2002, leishmaniasis cases were estimated to be 200,000 in Kabul [67]. Most of the outbreaks reported were anthroponotic cutaneous leishmaniasis and zoonotic cutaneous leishmaniasis originating from various regions of Afghanistan between 1987 and 2006 [68], so Afghan immigrants with leishmaniasis could be major reservoirs of leishmaniasis in Iran.

Due to laboratory facility limitations, the real scope of the threat of hepatitis B in Afghanistan is not at all clear; therefore, the prevalence of hepatitis B may be underestimated in Afghanistan. However, it can be assumed that around 10% of Afghans are infected with hepatitis B [69]. Only one study [27] that met the criteria for inclusion in this review addressed the proportion of hepatitis B patients who were Afghan. According to that study, 14% of patients with hepatitis B were Afghan immigrants. The prevalence of hepatitis B virus infection among Afghan immigrants was significantly higher than that among Iranians (*p*= 0.046). The prevalence of hepatitis B in Afghan immigrants in Iran may be high due in particular to the greater prevalence of injection drug use among Afghan immigrants [70]. This may be an important reservoir of hepatitis B in Iran. In general, injection drug use is one of the main pathways by which hepatitis B spreads [69].

There have been several educational workshops for social health workers, licensed health staff, and midwives to increase the health knowledge of more than three thousand Afghans living in Iran. It would seem advisable that such programs and similar educational plans be continued so as to increase the general knowledge of Afghan immigrants.

Although the studies reviewed in this article mainly discussed reported cases of different diseases among immigrant Afghans, it was rare to find studies that mentioned the incidence and prevalence of these diseases in this population and compared them with the Iranian population.

In this study, we only estimated the proportion of Afghan immigrants with certain infectious diseases (TB, MDR TB, malaria, CCHF, cholera, leishmaniasis and hepatitis B) in Iran. We could not estimate the proportion of Afghan immigrants in Iran with other infectious diseases such as HIV/AIDS and hepatitis C virus. Nor could we compare the prevalence of these diseases among the general Iranian population and the resident Afghan population in Iran. We did not have access to complete demographic information for the Afghan immigrants, such as age, education, or employment, so we could not interpret our results based on the demographic information of the Afghan immigrants.

## CONCLUSION

The overall pooled estimated proportion of Afghan immigrants in Iran with TB, MDR TB, malaria, CCHF, cholera, leishmaniasis, and hepatitis B was 29%. However, it seems that this estimate was lower than the true proportion. Considering the long common border between Iran and Afghanistan and the high prevalence of travel between these two countries, it is crucial to develop and equip surveillance centers on the borders for early diagnosis of suspicious cases of infections and also for the prevention of the distribution of disease from these locations to other points in Iran. During epidemics of infectious diseases in Afghanistan, careful attention should be paid to the entry points and regulations should be strengthened for examining those people intending to enter Iran from Afghanistan.

In general, more studies on knowledge, attitude and practice among Afghan nationals and a comparison between them and the Iranian population with regard to infectious disease is absolutely necessary to gain enough evidence for better interventions.

## Figures and Tables

**Table 1.** Characteristics of the included studies in meta-analysis

Author (published year) [Ref]	Location	Type of the disease	Sex	Sample size	Afghan patients (%)	Iranian patients (%)
Abbasi et al. (2004) [17]	Gorgan	Leishmaniasis	M, F	175	2.29	97.71
Alavi-Naini et al. (2006) [18]	Zabol & Zahedan	CCHF	M, F	255	25.5	74.5
Aminzadeh & Akhyani (2005) [19]	Tehran	Tuberculosis	M, F	81	17.28	82.72
Amirmozafari et al. (2006) [20]	Tehran	Tuberculosis	M, F	439	21.18	78.82
Ayatollahi et al. (2010) [21]	Yazd	Tuberculosis	M, F	32	21.88	78.12
Ayatollahi et al. (2010) [21]	Shiraz	Tuberculosis	M, F	104	45.2	54.8
Barati et al. (2010) [22]	Karaj	Cholera	M, F	54	7.4	92.6
Doroudchi et al. (2000) [23]	Shiraz	Tuberculosis	M, F	101	20.79	79.2
Ebrahimzadeh et al. (2009) [24]	Birjand	Tuberculosis	M, F	840	3.93	96.07
Farnia et al. (2006) [25]	Tehran	MDR TB	M, F	263	66.54	33.46
Farnia et al. (2006) [25]	Tehran	Tuberculosis	M, F	1479	33.34	66.66
Faenia et al. (2008) [26]	Tehran	Tuberculosis	M, F	199	22.61	77.39
Fathimoghaddam et al. (2011) [27]	Mashhad	Hepatitis B	M, F	22	13.64	86.36
Forotani (2007) [28]	Larestan	Malaria	M, F	80	33.75	66.25
Izadi et al. (2004) [29]	Zahedan & Zabol	CCHF	M, F	24	20.8	79.2
Jonaidi et al. (2007) [30]	National	Cholera	M, F	1115	2.24	97.76
Kadivar et al. (2007) [31]	Shiraz	Tuberculosis	M, F	1026	36.16	63.84
Khalifesoltani et al. (1997) [32]	Kashan	Tuberculosis	M, F	183	32.79	67.21
Khatami et al. (2008) [33]	Tehran	Tuberculosis	M, F	30	36.67	63.33
Khazaei et al. (2005) [35]	Zabol	Cholera	M, F	362	18.78	81.22
Khazaei et al. (2005) [34]	Zabol	Tuberculosis	M, F	2731	23.51	76.49
Mansoori et al. (2003) [36]	National	Tuberculosis	M	373	31.5	68.49
Mohammadi Anzi et al. (2008) [37]	Damghan	Tuberculosis	M, F	89	222.47	77.53
Mohammadi Anzi et al. (2010) [38]	Damghan	Leishmaniasis	M, F	465	4.3	95.7
Momeni & Aminjavaheri (1994) [39]	Isfahan	Leishmaniasis	M, F	1250	13.2	86.8
Moshfe et al. (2003) [40]	Kohgiluyeh & Boyer-Ahmad	Malaria	M, F	485	37.32	62.68
Mousavi et al. (2009) [41]	Kashan	Tuberculosis	M, F	196	54.59	45.81
Najafi et al. (2006) [42]	Mazandaran	Malaria	M, F	518	80.31	19.69
Raeisi et al. (2009) [43]	National 2002	Malaria	M, F	15378	42.13	57.87
Raeisi et al. (2009) [43]	National 2003	Malaria	M, F	25027	21.9	78.1
Raeisi et al. (2009) [43]	National 2004	Malaria	M, F	13166	30.49	69.51
Raeisi et al. (2009) [43]	National 2005	Malaria	M, F	19285	13.5	86.5
Raeisi et al. (2009) [43]	National 2006	Malaria	M, F	15896	10.39	89.61
Raeisi et al. (2009) [43]	National 2007	Malaria	M, F	16647	7.45	92.55
Ramazanzadeh et al. (2006) [44]	National	Tuberculosis	M, F	345	25.22	74.78
Ranjbar et al. (2010) [45]	Karaj	Cholera	M, F	110	4.55	95.45
Salarri & Kalantari (2004) [46]	Yazd	Tuberculosis	M, F	600	36.34	63.66
Salehi & Pourahmad (2001) [47]	Isfahan	Tuberculosis	M, F	164	45.12	54.88
Setoudeh Maram & Fararoei (1999) [48]	Fars	Tuberculosis	M, F	977	13.2	86.79
Shamaei et al. (2009) [49]	National	Tuberculosis	M, F	548	33.94	66.06
Sharifi-Mood (2006) [57]	Zahedan	Tuberculosis	M, F	195	16.92	83.08
Soleimanifard et al. (2011) [51]	Isfahan	Malaria	M, F	675	97.92	3.55
Tajadin et al. (2008) [52]	Tehran	Tuberculosis	M, F	191	38.22	61.78
Velayati et al. (2009) [53]	National	MDR TB	M, F	146	44.52	55.47
Yazdanpanah et al. (2003) [54]	Tehran	Tuberculosis	F	481	27.65	72.35
Yazdanpanah et al. (2003) [54]	Tehran	Tuberculosis	M	547	35.28	64.72
Youssefi & Taghi (2011) [55]	Sistan & Baluchestan	Malaria	M, F	1461	3.35	82.68
Zia-Sheikholeslami & Rezaeian (2010) [56]	Rafsanjan	Malaria	M, F	742	98.92	1.08

Ref, reference; CCHF, Crimean-Congo hemorrhagic fever; MDR TB, multiple-drug-resistant tuberculosis; M, male; F, female.

**Table 2.** Subgroup analysis of proportion of Afghan immigrant population with infectious diseases by quality of the included studies, year of studies conduction and type of infectious diseases

	Afghan immigrants		p-value^1^
	Proportion	95% CI	
Quality of included studies			
Low risk	0.206	0.146, 0.267	0.001
Intermediate	0.305	0.218, 0.392	0.001
High risk	0.354	0.108, 0.601	0.001
Year of study conduction			
1994-2000	0.118	0.132, 0.244	0.001
2000-2007	0.307	0.221, 0.393	0.001
2007-2011	0.277	0.053, 0.607	0.001
Type of diseases			
Tuberculosis	0.288	0.233, 0.344	0.001
MDR TB	0.557	0.341, 0.773	0.001
Malaria	0.398	0.230, 0.566	0.001
Cholera	0.082	0.004, 0.159	0.001
Crimean Congo Hemorrhagic fever	0.25	0.200, 0.301	0.59
Leishmaniasis	0.066	0.000, 0.132	0.001

CI, confidence interval; MDR TB, multiple-drug-resistant tuberculosis.

1 p-value for chi square test for heterogeneity.
